# Hepatocyte ALOXE3 is induced during adaptive fasting and enhances insulin sensitivity by activating hepatic PPAR**γ**

**DOI:** 10.1172/jci.insight.120794

**Published:** 2018-08-23

**Authors:** Cassandra B. Higgins, Yiming Zhang, Allyson L. Mayer, Hideji Fujiwara, Alicyn I. Stothard, Mark J. Graham, Benjamin M. Swarts, Brian J. DeBosch

**Affiliations:** 1 Department of Pediatrics and; 2 Department of Medicine, Diabetic Cardiovascular Disease Center, Washington University School of Medicine, St. Louis, Missouri, USA.; 3 Department of Chemistry & Biochemistry, Central Michigan University, Mt. Pleasant, Michigan, USA.; 4 IONIS Pharmaceuticals, Carlsbad, California, USA.; 5 Department of Cell Biology & Physiology, Washington University School of Medicine, St. Louis, Missouri, USA.

**Keywords:** Hepatology, Metabolism, Diabetes, Insulin signaling, Signal transduction

## Abstract

The hepatic glucose fasting response is gaining traction as a therapeutic pathway to enhance hepatic and whole-host metabolism. However, the mechanisms underlying these metabolic effects remain unclear. Here, we demonstrate the epidermal-type lipoxygenase, eLOX3 (encoded by its gene, *Aloxe3*), is a potentially novel effector of the therapeutic fasting response. We show that *Aloxe3* is activated during fasting, glucose withdrawal, or trehalose/trehalose analogue treatment. Hepatocyte-specific *Aloxe3* expression reduced weight gain and hepatic steatosis in diet-induced and genetically obese (*db/db*) mouse models. *Aloxe3* expression, moreover, enhanced basal thermogenesis and abrogated insulin resistance in *db/db* diabetic mice. Targeted metabolomics demonstrated accumulation of the PPAR*γ* ligand 12-KETE in hepatocytes overexpressing *Aloxe3*. Strikingly, PPAR*γ* inhibition reversed hepatic *Aloxe3*–mediated insulin sensitization, suppression of hepatocellular ATP production and oxygen consumption, and gene induction of PPAR*γ* coactivator-1*α* (PGC1*α*) expression. Moreover, hepatocyte-specific PPAR*γ* deletion reversed the therapeutic effect of hepatic *Aloxe3* expression on diet-induced insulin intolerance. *Aloxe3* is, therefore, a potentially novel effector of the hepatocellular fasting response that leverages both PPAR*γ*-mediated and pleiotropic effects to augment hepatic and whole-host metabolism, and it is, thus, a promising target to ameliorate metabolic disease.

## Abstract 2

The lipoxygenase ALOXE3 is an effector of the hepatic fasting response that improves insulin sensitivity by activating hepatic PPARγ.

## Graphical abstract

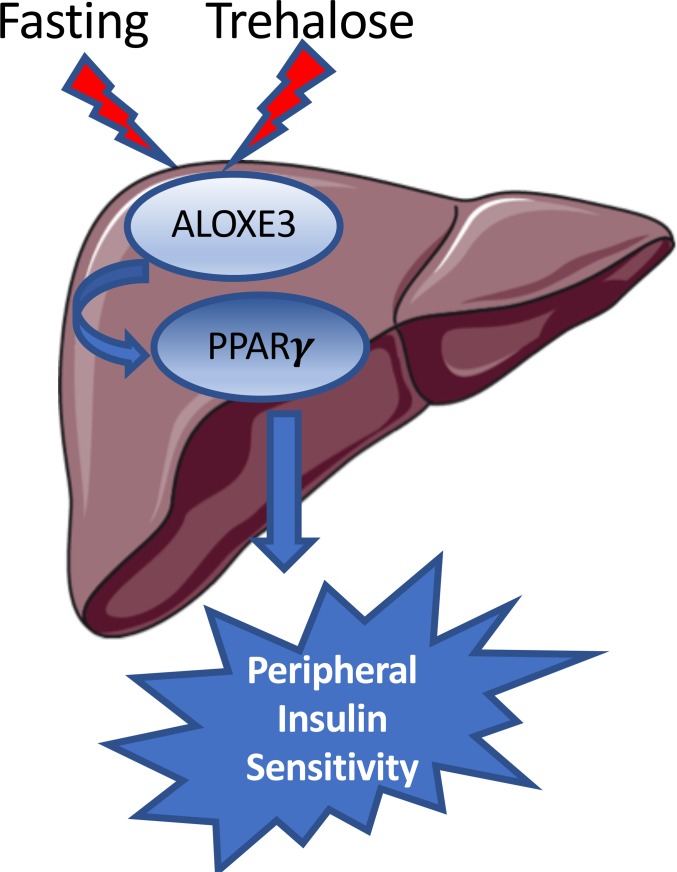

## Introduction

Leveraging the generalized fasting and caloric restriction responses to mitigate metabolic disease is under intense investigation as a novel therapeutic approach against obesity, diabetes mellitus, and nonalcoholic fatty liver disease (NAFLD) (1, 2). The hepatocyte is uniquely positioned at the nexus of the portal circulation — where dietary macronutrients are sensed and triaged to their metabolic fate — and the peripheral circulation, from which macronutrients are taken up and utilized or stored. Its unique anatomic positioning also defines the hepatocyte’s role in coordinating the transition from fed to fasting states. Specifically, the hepatocyte senses fat, protein, and carbohydrate content and rapidly alters its metabolism and its endocrine functions to coordinate peripheral responses. However, the therapeutic promise of caloric restriction and generalized macronutrient fasting remains limited by its clinical impracticality and unsustainability. Our laboratory and others (1, 9, 11) have therefore examined specific pathways through which fasting and fasting-like mechanisms can be molecularly or pharmacologically leveraged to the benefit of the host.

Upon physiological macronutrient withdrawal, the canonical hepatic response is to shift to mobilize glycogen and oxidize fat from stores to produce glucose and ketone body fuel for the brain and heart. This begins with activation of proximal macronutrient sensing kinases, AMPK, and mTOR. These kinases act as primary regulators of macroautophagy as well as transcriptional fasting programming by hepatic transcription factors, including transcription factor EB (3); PPARγ coactivator-1α (PGC1α) (4); and transcriptional modifiers such as the sirtuin family of protein deacetylases (5). FGF21 is released from the liver of fasting rodents and humans to promote adipose “browning” (mitochondrial uncoupling to promote heat generation; ref. 6). The net effect of this signaling initiation is a networked cascade through which the liver provides energy substrate to the periphery during fasting and promotes peripheral insulin sensitivity for efficient absorption of the next meal.

We recently demonstrated that blocking hepatic glucose transporters genetically or pharmacologically closely recapitulates the therapeutic metabolic effects of generalized macronutrient fasting (7–11). Pharmacologic glucose transport blockade using the disaccharide glucose transporter inhibitor trehalose blocked mTOR and activated AMPK-dependent autophagic flux (9, 12). Moreover, trehalose blocked diet- and genetically induced steatosis via ATG16L1- and AMPK-dependent mechanisms, and it enhanced peripheral thermogenesis via hepatocyte TFEB- and FGF21-dependent mechanisms (11). Consistent with data from pharmacological glucose transporter (GLUT) blockade, genetic deletion of the hepatocyte GLUT8 similarly increased basal and fasting heat generation (10), enhanced hepatocyte fat oxidation, and inhibited hepatic de novo lipogenesis and triacylglycerol generation in mice fed a high-fructose diet (7, 8). Notably, in those experiments, GLUT8 blockade was associated with enhanced insulin tolerance, enhanced hepatic PPARγ expression, and concomitant activation of the PPARγ target UCP2 (8). Conversely, GLUT8 was upregulated in the livers of genetically diabetic mice (13). Together, prior data suggest that pharmacologic and genetic activation of hepatic glucose fasting–like responses are sufficient to mitigate metabolic disease. However, the mechanisms underlying this protection remain enigmatic.

Recent data illuminate a role for cellular lipoxygenases in the pathogenesis and protection from metabolic disease. In humans, a recent large cohort of adults with type 2 diabetes demonstrated a potentially protective role for 5-lipoxygenases ALOX5 and ALOX5AP against diabetes mellitus (14). Conversely, ALOX12/15 inhibition in rodent models inhibited oxidative stress, β cell deterioration, hepatic steatosis, and systemic inflammation (15–18). Together, these data suggest that intracellular lipid intermediary metabolism drives both tissue-level and whole-organism metabolic homeostasis. The concept that the fasting state might modulate lipoxygenase activity as part of the broadly adaptive effects of fasting on metabolism remained heretofore unexplored.

Here, we identified eLOXE3 (encoded by the *Aloxe3* gene) as a fasting-induced epidermal-type lipoxygenase that acts as a potentially novel effector of the hepatic glucose fasting response in vitro and in vivo. Targeted *Aloxe3* overexpression induced robust generation of the PPARγ ligand (19, 20) 12-KETE. In vivo, hepatic *Aloxe3* expression enhanced insulin sensitivity, attenuated weight gain, and reduced hepatic steatosis in both diet- and genetically induced steatosis models. Moreover, hepatic *Aloxe3* expression mitigated diet-induced dyslipidemia, and *db/db* mice expressing *Aloxe3* exhibited attenuated weight gain, enhanced basal caloric expenditure, and PPARγ-dependent insulin sensitivity. Strikingly, we demonstrate that hepatocyte-specific PPARγ deletion is absolutely required for the insulin-sensitizing effect of hepatic *Aloxe3* induction. Taken together, *Aloxe3* is a fasting-responsive hepatocyte effector that is sufficient to attenuate insulin resistance, weight gain, and hepatic fat deposition in dietary and genetically obese models, in part by activating hepatic PPARγ. *Aloxe3* is, thus, a novel therapeutic mechanism downstream of the fasting response, which can be leveraged against metabolic disease.

## Results

**Figure 1:**
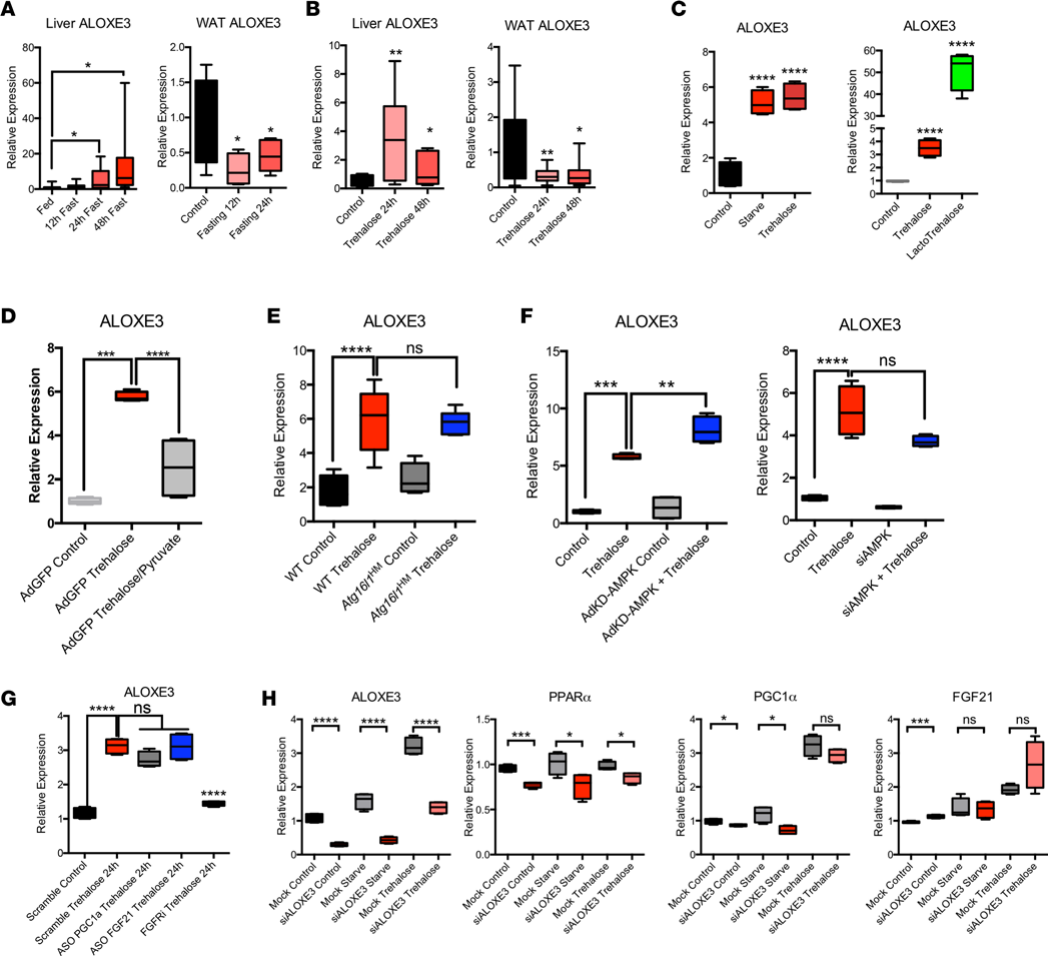
*Aloxe3* is induced in response to fasting and pseudofasting. (**A**) *Aloxe3* expression in liver and white adipose tissue (WAT) from fasting WT mice (0–48 hours [h]). *n* = 6 each of fed, 12 h fasting, and 24 h fasting mice. For 48 h fasting mice, *n* = 7. (**B**) *Aloxe3* expression in WT mice fed oral trehalose (3% in sterile water fed ad libitum, 0–48 h). For control and trehalose (24 h) mice, *n* = 5 and 6. For 48 h trehalose-treated mice, *n* = 6. (**C**) *Aloxe3* expression in isolated primary murine hepatocytes treated with 0.5% FCS/1 g/l glucose media, 100 mM trehalose, or 100 mM lactotrehalose (24 h). *n* = 4 per group. (**D**) *Aloxe3* expression in trehalose-treated isolated primary hepatocytes pretreated with or without 5 mM pyruvate. *n* = 4 per group. (**E**) *Aloxe3* expression in trehalose-treated WT and *Atg16l1*-mutant mice in vivo (24 h). *n* = 6 per group. (**F**) *Aloxe3* expression in response to trehalose in the presence or absence of kinase-dead AMPK overexpression or siRNA-mediated AMPK knockdown. *n* = 4 per group. (**G**) *Aloxe3* expression in response to trehalose in the presence or absence of antisense oligonucleotide (ASO) directed against PGC1, or FGF21. FGF receptor 1–4 inhibitor (LY2874455) was included as a control to demonstrate *Aloxe3* blockade in context. *n* = 4 per group. (**H**) Fasting-responsive marker gene expression in the presence or absence of trehalose following treatment with or without *Aloxe3*-directed siRNA. *P < 0.05, **P < 0.01, ***P < 0.001, and ****P < 0.0001 by 2-tailed *t* test with Bonferroni-Dunn post hoc correction versus untreated controls group or versus the bracketed comparison group where indicated.

To test the hypothesis that *Aloxe3* is induced by acute fasting or pseudo-fasting conditions, we subjected WT mice to 48-hour fasting. During acute fasting, hepatic *Aloxe3* expression was increased and sustained at least through 48 hours (Figure 1A). Oral administration of the fasting-mimetic hepatic glucose transport inhibitor trehalose (3% PO, ad libitum), similarly induced *Aloxe3* expression after 24-hour and 48-hour trehalose feeding (Figure 1B). The hepatic *Aloxe3* response to fasting contrasted with fasting *Aloxe3* expression in white adipose tissue (WAT), wherein *Aloxe3* mediates fat differentiation and lipid storage. Accordingly, *Aloxe3* was significantly suppressed during both fasting and trehalose feeding in epididymal WAT (Figure 1, A and B, respectively). We then modeled *Aloxe3* expression in vitro to determine whether fasting-induced *Aloxe3* expression is cell-autonomously regulated. Isolated primary murine hepatocytes were subjected to treatment by either low serum and glucose (0.5% serum, 1 g/l glucose) or trehalose (Figure 1C). In addition, we characterized fasting-mimetic effects of a trehalose analogue that resists enzymatic degradation by trehalases (21–23), called lactotrehalose (α-D-glucopyronosyl-(1,1)-α-D-galactopyranoside; Figure 1C and Supplemental Figure 1; supplemental material available online with this article; https://doi.org/10.1172/jci.insight.120794DS1). In each case, *Aloxe3* expression was significantly increased, most potently by trehalose and lactotrehalose (Figure 1C and Supplemental Figure 1).

In light of evidence that *Aloxe3* is a potentially novel hepatocyte fasting-responsive factor in mice and in isolated murine hepatocytes, we evaluated whether this factor is expressed in isolated human hepatocytes and in Huh7 and HepG2 human hepatoma cell lines. Consistent with immunoblot and immunohistochemical eLOX3 protein detection in human liver tissue (24), quantitative PCR (qPCR) analysis demonstrated *ALOXE3* expression in both HepG2 and in primary human hepatocytes (Supplemental Figure 2).

To determine whether *Aloxe3* induction depended upon energy substrate deficit, we treated primary hepatocytes with trehalose in the presence or absence of pyruvate. This provided energy substrate for the cell, independently of glucose transporter blockade. Pyruvate reversed trehalose-induced *Aloxe3* induction by ~60% (*P* < 0.0001), suggesting that *Aloxe3* expression is at least partly energy substrate dependent (Figure 1D). In light of our prior data in which we demonstrated that the autophagy complex protein ATG16L1 was required for autophagic and antisteatotic effects of trehalose in hepatocytes (9, 25), we examined whether ATG16L1 is required for *Aloxe3* induction. We treated primary hepatocytes from WT littermates or from mice with homozygous hypomorphic *Atg16l1* alleles (Atg16l1^HM^) with or without trehalose (24 hours). *Aloxe3* mRNA quantification revealed robust *Aloxe3* induction in WT hepatocytes, which was not suppressed in *Atg16l1^HM^* hepatocytes (Figure 1E). Similarly, AMPK inhibition (by kinase-dead AMPK overexpression or by *Aloxe3* siRNA transfection; Figure 1F) and PPARα, PGC1α, and FGF21 knockdown each failed to reverse *Aloxe3* induction after trehalose treatment or glucose and serum withdrawal (Figures 1G and 1H). Accordingly, moderate hepatocyte-specific overexpression of the fasting-induced transcription factor SIRT1 did not induce *Aloxe3* expression (Supplemental Figure 3). In contrast, siRNA-based *Aloxe3* knockdown attenuated PPARα and PGC1α at baseline and during glucose and serum withdrawal from hepatocytes in vitro, without effects on FGF21 expression (Figure 1H). Together, our data demonstrate that *Aloxe3* is a hepatocyte lipoxygenase that is induced by glucose transporter blockade and energetic deficit via a mechanism that does not require canonical ATG16L1-dependent and AMPK-PGC1α/PPARα-FGF21 fasting mechanisms.

**Figure 2:**
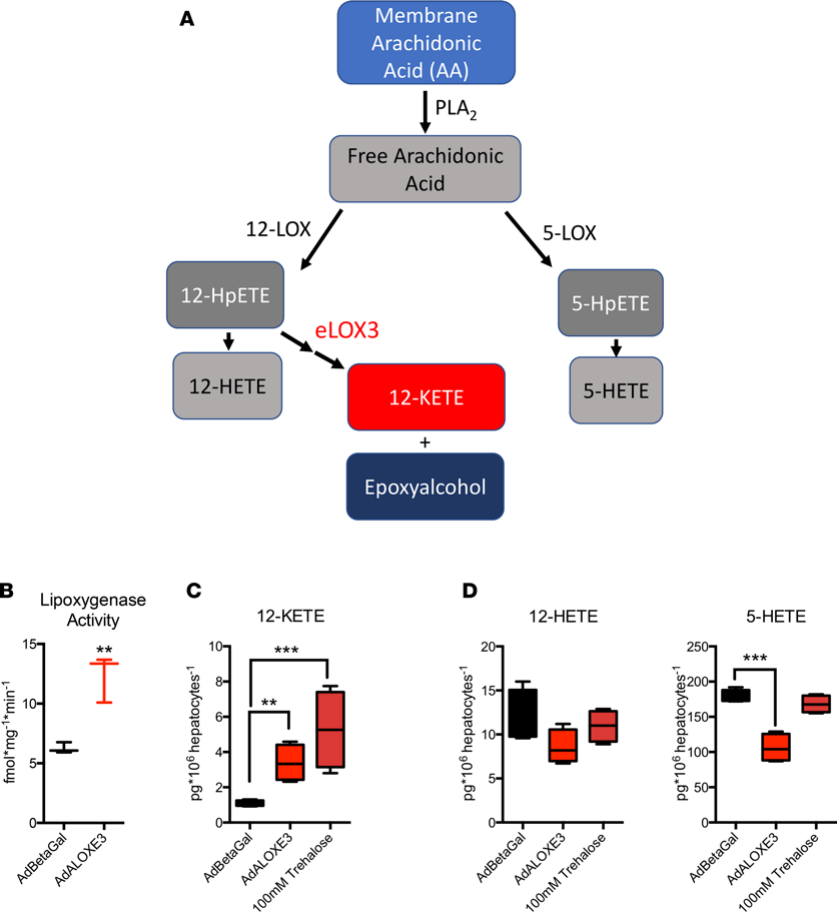
*Aloxe3* and trehalose induce the PPARγ ligand 12-KETE in murine hepatocytes. (**A**) Arachidonic acid metabolism mediated by lipoxygenases 12-LOX, 5-LOX, and eLOX3 (adapted from refs. 26, 29, 58, 59). (**B**) Lipoxygenase activity in primary murine hepatocytes upon overexpression of β galactosidase or *Aloxe3*. *n* = 3 from 1 representative experiment of 2 experiments with similar results. (**C** and **D**) Quantitative GC-MS analysis of the stable lipoxygenase reaction products 12-KETE, 5-HETE, and 12-HETE. Data represent 4 independent cultures per group from 1 experiment, representing 2 independent experiments with similar results. **P < 0.01 and ***P < 0.001 by 2-tailed *t* test with Bonferroni-Dunn post hoc correction versus untreated controls group or versus the bracketed comparison group where indicated.

eLOX3 generates the PPARγ ligand 12-KETE and an epoxyalcohol from 12-HpETE in the metabolism of plasma membrane arachidonic acid (Figure 2A) (26–30). We tested whether *Aloxe3* mediates similar lipid metabolism in hepatocytes upon forced *Aloxe3* expression. We first evaluated the effect of *Aloxe3* overexpression on hepatocyte lipoxygenase activity by measuring LOX substrate oxidation in cell extracts from hepatocytes overexpressing β galactosidase (β-gal) or *Aloxe3*. Consistent with prior data demonstrating intrinsic, latent dioxygenase activity of eLOX3 (31), *Aloxe3* overexpression significantly increased total oxidized LOX substrate accumulation (Figure 2B) and 12-KETE accumulation in primary hepatocytes (Figure 2C). This effect of *Aloxe3* overexpression on 12-KETE accumulation was indeed phenocopied by trehalose treatment (Figure 2C). Moreover, *Aloxe3* overexpression decreased the abundance of alternate arachidonic acid metabolic pathway products 5-HETE and 12-HETE (Figure 2D).

**Figure 3:**
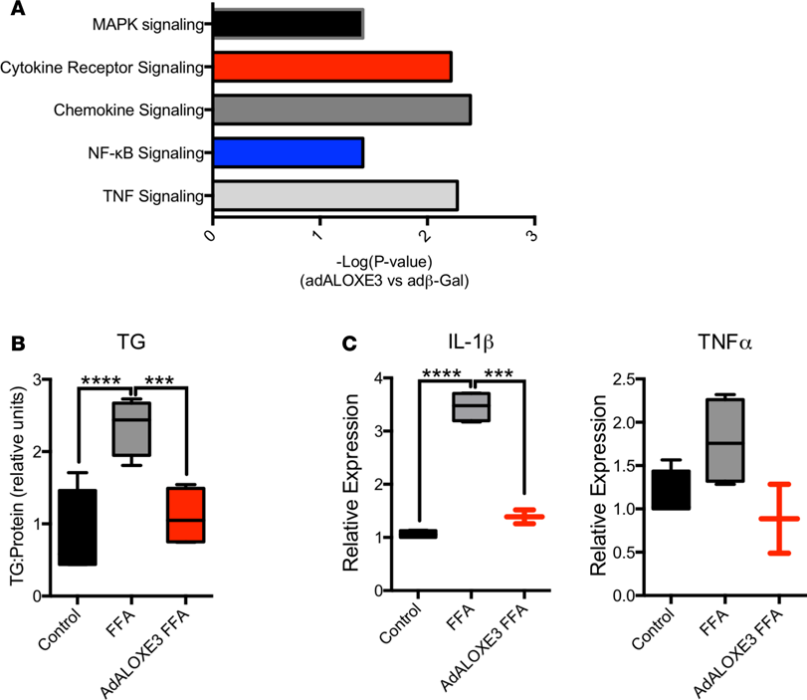
*Aloxe3* reduces inflammatory signaling and steatosis in hepatocytes. (**A**) RNA-seq analysis of the top 9 downregulated genes in primary hepatocytes expressing either β galactosidase or *Aloxe3*. *n* = 3 from a single RNA-seq run performed once. The *P* value for significantly downregulated pathways is demonstrated as (–Log[*P* value]). (**B**) Hepatic TG accumulation and (**C**) IL-1β or TNFα expression in hepatocytes treated with BSA-conjugated fatty acids with or without *Aloxe3* expression. *n* = 4 in **B** and *n* = 4 in **C**, single representative experiments, which were repeated twice with similar results. ***P < 0.001 and ****P < 0.0001 by 2-tailed *t* testing with Bonferroni-Dunn post hoc correction versus the bracketed comparison group as indicated.

To ascertain transcriptome-wide effects of forced *Aloxe3* overexpression, we next treated primary hepatocytes with adenovirus encoding β-gal or *Aloxe3* prior to RNA sequencing (RNA-seq) analysis. Pathway analysis revealed that 5 of the 10 most downregulated processes in *Aloxe3*-overexpressing cells were devoted to inflammatory signaling, including TNFα, NF-κB, chemokine signaling, cytokine receptor signaling, and MAPK signaling (Figure 3A). Given these findings, and in light of the fact that *Aloxe3* upregulation by trehalose correlates with reduced diet- and genetically induced steatosis (9, 25), we examined whether *Aloxe3* attenuates fat-induced inflammatory signaling and triglyceride (TG) accumulation. Primary hepatocytes treated with BSA-conjugated fatty acids (FA) induced *Il1b* and *Tnfa* gene expression, as well as TG accumulation (Figure 3, B and C). These FA-induced effects on TG accumulation and *Il1b* and *Tnfa* gene expression effects were reversed in FA-treated cultures overexpressing *Aloxe3*.

**Figure 4:**
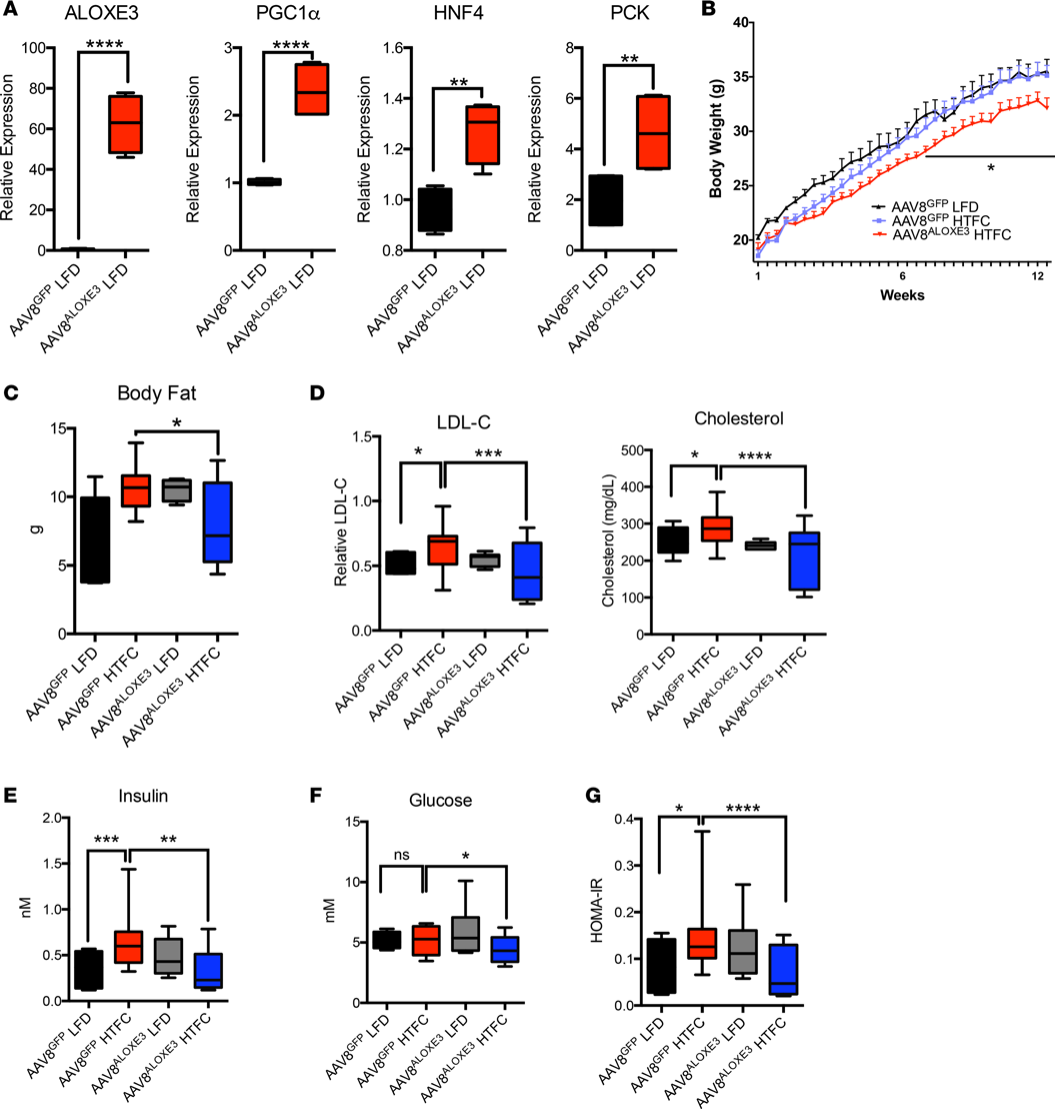
Enhanced whole-body metabolism in mice ALOXE3-overexpressing mice. (**A**) qPCR quantification of expression for oxidative and fasting-response genes in unperturbed mice expressing hepatocyte GFP or *Aloxe3*. (**B**) Body weight over time in low-fat or high–trans fat/cholesterol diet–fed mice expressing hepatocyte GFP or *Aloxe3*. (**C**) Body fat content in mice fed HTFC or LFD with or without hepatic *Aloxe3* overexpression. (**D**) LDL-C and total cholesterol in mice fed HTFC or LFD with or without hepatic *Aloxe3* overexpression. (**E–G**) Circulating insulin, glucose, and calculated HOMA-IR in LFD- and HTFC-fed mice overexpressing hepatocyte GFP or *Aloxe3*. Number of mice in each group is: 5, AAV8^GFP^ LFD; 10, AAV8^GFP^ HTFC; 5, AAV8^ALOXE3^ LFD; and 10, AAV8^ALOXE3^ HTFC. *P < 0.05, **P < 0.01, ***P < 0.001, and ****P < 0.0001 by 2-tailed *t* test with Bonferroni-Dunn post hoc correction versus the bracketed comparison group as indicated.

*Aloxe3*-deficient mice are not amenable to in vivo metabolic studies because germline *Aloxe3* deficiency results in postnatal mortality secondary to massive skin permeability and water loss (32). To evaluate the in vivo metabolic consequences of hepatocyte *Aloxe3* activation, we tested the in vivo effects of hepatocyte-directed *Aloxe3* expression. We first confirmed overexpression of the *Aloxe3* transgene in liver (Figure 4A) without changes in *Aloxe3* expression in skeletal muscle, WAT, or brown adipose tissue (not shown). This correlated with upregulation of fasting and oxidative genes in unperturbed *Aloxe3*-overexpressing mice, including *Aloxe3* (Figure 4A), *Ppargc1a* (PGC1), hepatocyte nuclear factor 4α (*Hnf4A/*HNF4α), and phosphoenolpyruvate carboxykinase 1 (*Pck1*, Figure 4A.).

We next evaluated the effect of hepatic *Aloxe3* expression in mice fed low-fat diet (LFD) or steatogenic, high trans-fat/cholesterol diet (HTFC) (12 weeks). Mice overexpressing *Aloxe3* fed HTFC gained significantly less weight, had lower total body mass (Figure 4B), and had lower body fat mass (Figure 4C) without changes in lean mass (not shown) when compared with *Gfp*-overexpressing mice fed HTFC. Accordingly, low density lipoprotein-cholesterol (LDL-C) and total cholesterol were significantly lowered in HTFC-fed mice overexpressing *Aloxe3* (Figure 4D). Indices of glucose homeostasis were also improved by *Aloxe3* overexpression in HTFC-fed mice (Figure 4, E–G). Specifically, HTFC feeding increased circulating insulin and homeostatic model assessment– insulin resistance (HOMA-IR) in GFP-overexpressing mice (Figure 4, E and G), without altering fasting plasma glucose (Figure 4F). In contrast, HTFC-fed mice overexpressing hepatic *Aloxe3* were protected from HTFC-induced hyperinsulinemia and insulin resistance (Figure 4, E and G). Indeed, fasting glucose was also lowered in *Aloxe3*-overexpressing, HTFC-fed mice when compared with *GFP*-expressing, HTFC-fed mice (Figure 4F). Together, hepatocyte *Aloxe3* expression was sufficient to reduce diet-induced weight gain, body fat accumulation, dyslipidemia, and insulin resistance.

**Figure 5:**
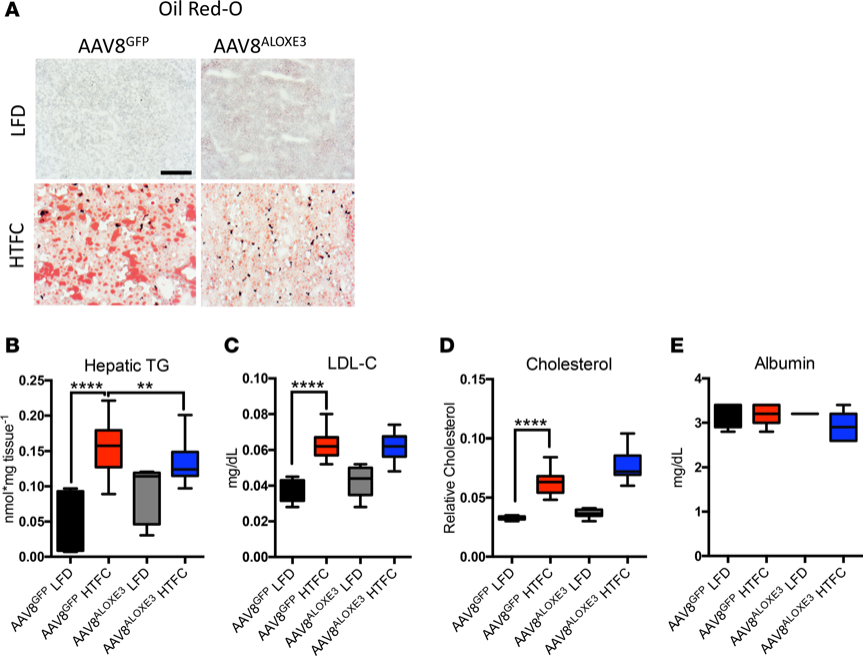
Reduced hepatic steatosis correlates with *Aloxe3*-induced fasting responses. (**A**) Oil Red O staining in livers from low-fat or high–trans fat/cholesterol–fed mice overexpressing *GFP* or *Aloxe3*. (**B–D**) Hepatic tissue quantification of triglycerides, LDL-C, total cholesterol in low-fat or high–trans fat/cholesterol–fed mice overexpressing empty vector or *Aloxe3*. (**E**) Serum albumin measurements in mice analyzed in **A–D** Number of mice in each group is: 5, AAV8^GFP^ LFD; 10, AAV8^GFP^ HTFC; 5, AAV8^ALOXE3^ LFD; and 10, AAV8^ALOXE3^ HTFC. Scale bar: 100 μm. **P < 0.01 and ****P < 0.0001 by 2-tailed *t* test with Bonferroni-Dunn post hoc correction versus untreated controls group or versus the bracketed comparison group where indicated.

We next examined hepatic lipid metabolism in GFP- and *Aloxe3*-overexpressing mice fed LFD or HTFC. Frozen liver sections from HTFC-fed mice exhibited increased Oil Red O staining and macrosteatosis when compared with LFD-fed mice. Consistent with prior models of PPARγ activation (33–35), *Aloxe3* expression in LFD-fed mice resulted in a mild basal TG accumulation (Figure 5, A and B). However, we did not observe evidence of hepatic inflammation or fibrosis by histological analysis of H&E- or trichrome-stained liver sections in LFD-fed mice after 12 weeks *Aloxe3* overexpression (Supplemental Figure 4). Also consistent with prior reports on hepatic PPARγ activation, *Aloxe3* overexpression modestly but significantly protected from HTFC-induced TG accumulation that took on a microsteatotic staining pattern (Figure 5, A and B). No changes in hepatic LDL-C or total cholesterol were observed (Figure 5, C and D) upon hepatic *Aloxe3* overexpression in LFD- or HTFC-fed mice. None of our genetic or dietary manipulations had any effect on hepatic synthetic function, as ascertained by quantitative circulating albumin levels (Figure 5E).

**Figure 6:**
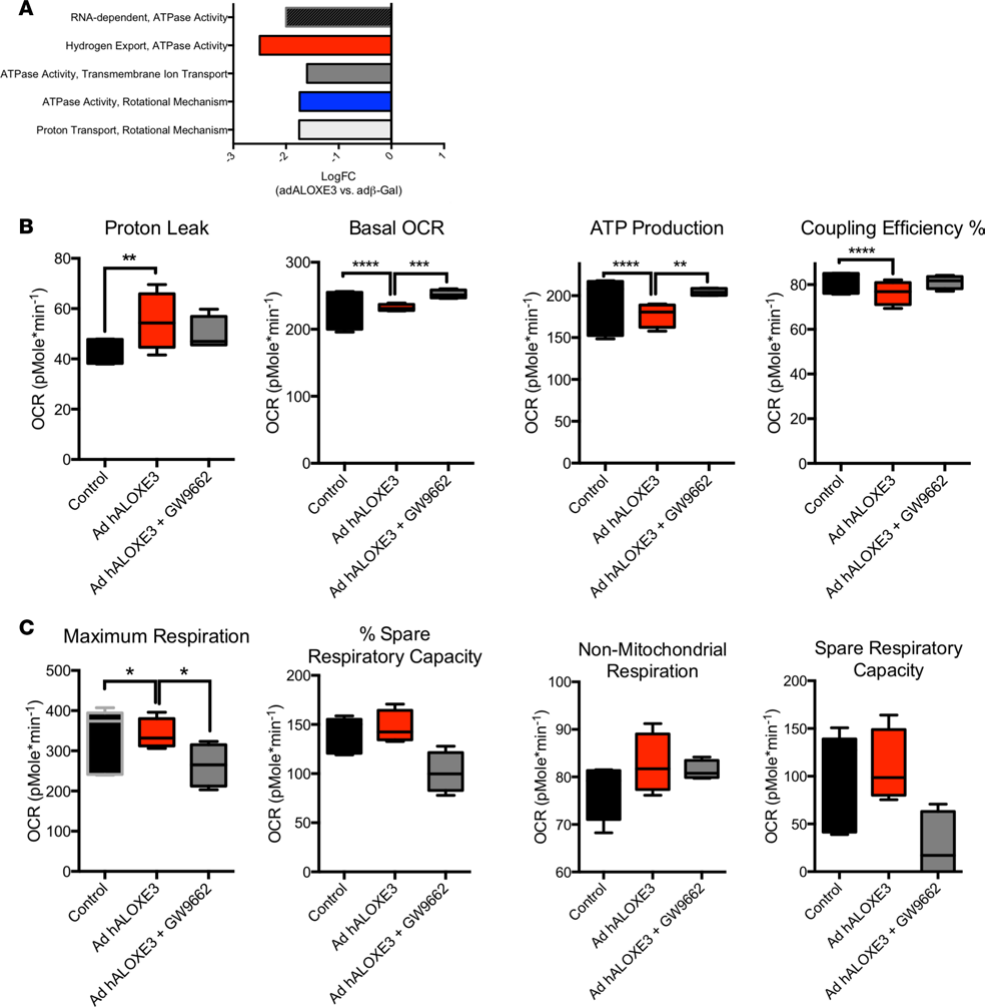
Hepatic PPARγ is required for *Aloxe3* to induce hepatic energetic inefficiency. (**A**) RNA-seq analysis of the top 5 downregulated molecular processes in hepatocytes overexpressing Aloxe3. Highlighted are ATPase-related and mitochondrial coupling processes. Graphed is logFC relative to cultures expressing β galactosidase. *n* = 3 from a single RNA sequencing run performed once. (**B** and **C**) Seahorse XF96 analysis of proton leak, basal OCR, ATP production, coupling efficiency, and nonmitochondrial oxygen consumption in AML12 cells overexpressing *Aloxe3* with or without GW9662 (PPARγ inhibitor) treatment. *n* = 8 independent cultures combined from 2 distinct experimental runs. *P < 0.05, **P < 0.01, ***P < 0.001, and ****P < 0.0001 by 2-tailed *t* testing with Bonferroni-Dunn post hoc correction versus the bracketed comparison group as indicated.

To examine mechanistically how *Aloxe3* might activate hepatocyte-starvation–like responses, we evaluated the effects of *Aloxe3* overexpression on mitochondrial respiratory function at the cellular and molecular levels. RNA-seq analysis of primary hepatocytes overexpressing *Aloxe3* revealed that 6 of the 10 most downregulated molecular processes encompassed mitochondrial electron transport function, including proton transport, ATPase activity, transmembrane ion transport, and hydrogen export (Figure 6A). We therefore tested functionally the hypothesis that *Aloxe3* mitigates ATP production by inducing hepatocyte mitochondrial uncoupling. Seahorse analysis of hepatocytes overexpressing *Aloxe3* exhibited elevated proton leak, ATP production, and coupling efficiency, concomitant with suppressed basal oxygen consumption rate and enhanced glycolytic rate when compared with hepatocytes expressing β-gal (Figure 6B). Each of these parameters was partly or fully reversed in the presence of the PPARγ inhibitor GW9662 (Figure 6B). No changes in nonmitochondrial oxygen consumption were observed (Figure 6C), suggesting that *Aloxe3* specifically affected mitochondrial energy metabolism.

**Figure 7:**
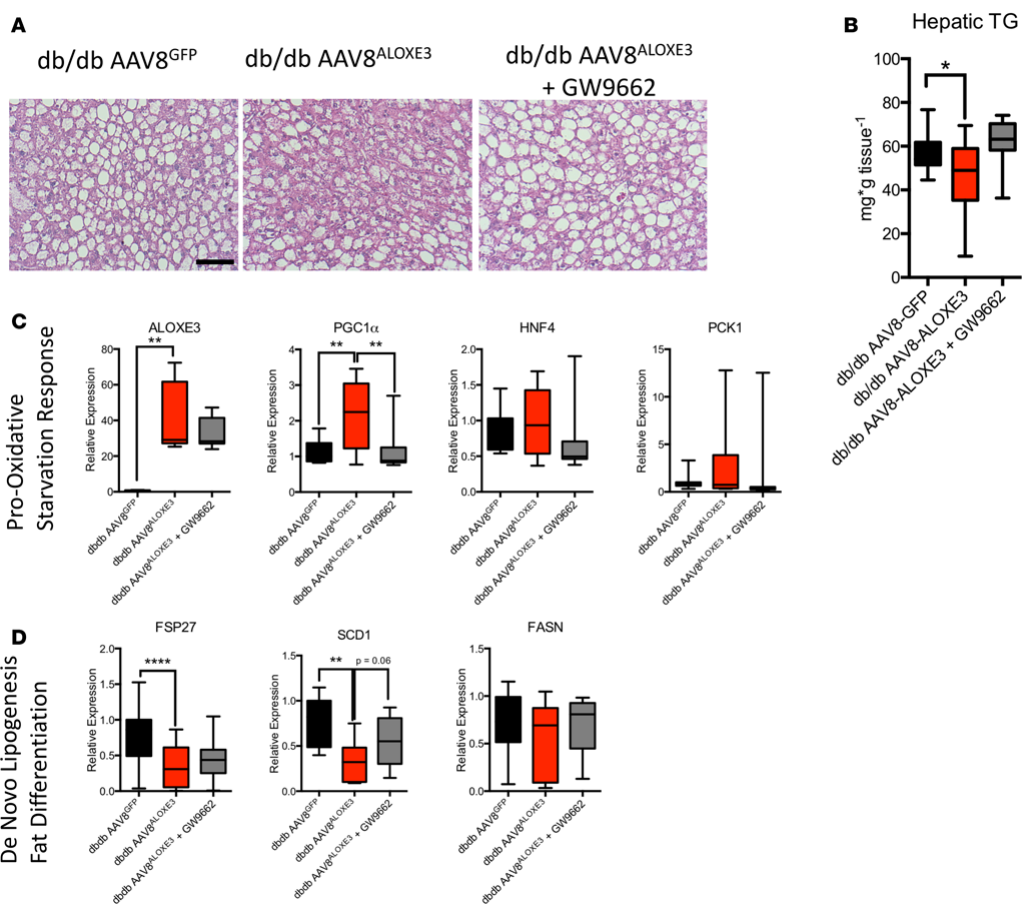
Reduced hepatic steatosis correlates with *Aloxe3*-induced fasting and reduction of de novo lipogenesis. (**A**) H&E staining in livers from low-fat or high–trans fat/cholesterol–fed mice overexpressing empty vector or *Aloxe3*. (**B**) Hepatic tissue triglyceride quantification in *db/db* mice overexpressing GFP or *Aloxe3* in the presence or absence of GW9662. (**C** and **D**) qPCR quantification of expression for oxidative and fasting-response genes (**C**) and de novo lipogenic genes (**D**) in *db/db* mice expressing GFP or *Aloxe3* with or without GW9662 administration. Number of mice in each group is: 10, *db/db* AAV8^GFP^; 10, *db/db* AAV8^ALOXE3^; 10, AAV8^ALOXE3^ + GW9662. Scale bar: 100 μm. **P < 0.01, or ****P < 0.0001 by 2-tailed *t* testing with Bonferroni-Dunn post hoc correction versus the bracketed comparison group as indicated.

Mechanistic interrogation in vivo was executed to determine whether hepatocyte *Aloxe3* mediated peripheral insulin and glucose homeostasis in a leptin-dependent manner. To that end, we overexpressed *Aloxe3* or *GFP* in *db/db* mice, which lack the leptin receptor. Again, in the liver, we observed modest, statistically significant attenuation of hepatic TG accumulation in *db/db* mice overexpressing *Aloxe3* when compared with *db/db* controls (Figure 7, A and B). We confirmed *Aloxe3* overexpression correlated with increases in oxidative and fasting-response genes *Ppargc1a*, *Hnf4a*, and *Pc1* (Figure 7C). Moreover, *Aloxe3* overexpression reduced genetic markers of de novo lipogenesis in *db/db* mice, including *Fsp27*, *Scd1*, and *Fasn* (Figure 7D). Concomitant PPARγ inhibition by the inhibitor GW9662 produced either statistically significant or trends toward significant reversal in each of these *Aloxe3*-altered marker genes (Figure 7, C and D).

**Figure 8:**
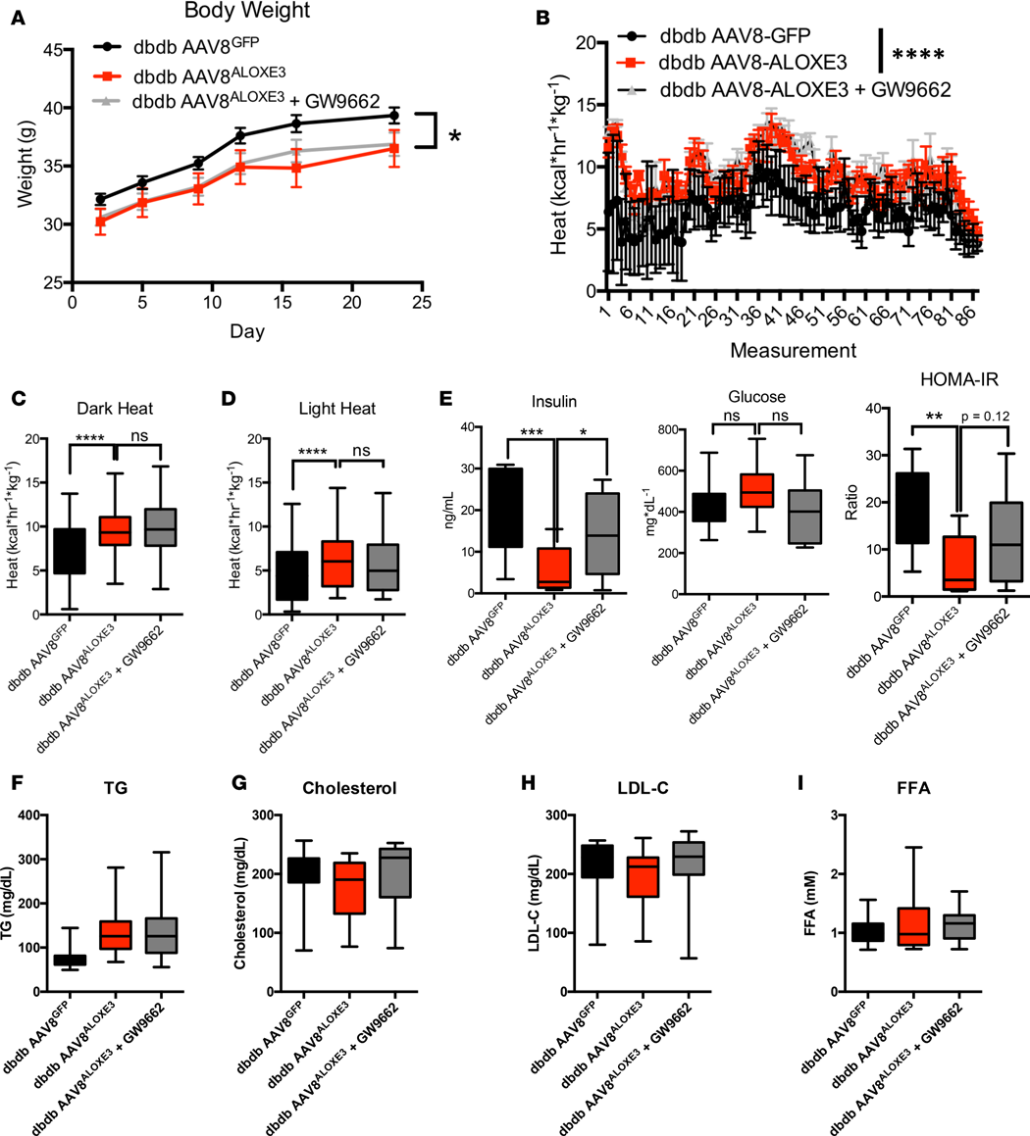
Enhanced whole-body energy metabolism in *db/db* diabetic mice overexpressing *Aloxe3*. (**A**) Body weight over time in *db/db* mice expressing empty vector or *Aloxe3* in the presence or absence of GW9662. (**B**) Heat generation over time in *db/db* mice expressing GFP or *Aloxe3*. (**C** and **D**) Indirect calorimetric quantification of light- and dark-cycle heat generation in *db/db* mice expressing *Aloxe3* or GFP treated with or without GW9662. (**E**) Fasting serum insulin determined by ELISA, serum glucose determined by colorimetric assay, and calculated HOMA-IR index based on glucose and insulin data. (**F–I**) Serum TG, cholesterol, LDL-C, and FFA content in *db/db* mice with or without hepatic *Aloxe3* overexpression and with or without GW9662 treatment. Number of mice in each group is: 10, *db/db* AAV8^GFP^; 10, *db/db* AAV8^ALOXE3^; 10, AAV8^ALOXE3^ + GW9662. *P < 0.05, **P < 0.01, ***P < 0.001, and < 0.0001 by 2-tailed *t* test with Bonferroni-Dunn post hoc correction versus bracketed comparison groups.

Although baseline weights were not statistically different, *db/db* mice overexpressing *Aloxe3* gained significantly less weight and had a lower end-of-trial weight than *db/db* mice over the 28-day trial (Figure 8A). Indirect calorimetry revealed that the attenuated weight gain in aloxe3 db/db mice was associated with enhanced light- and dark-cycle heat generation and O2-CO2 exchange (Figure 8, B-D). Neither heat generation nor O_2_-CO_2_ exchange in *Aloxe3^db/db^* was affected by GW9662 coadministration (Figure 8, B–D).

Because hepatic *Aloxe3* expression enhanced peripheral insulin sensitivity in HTFC-fed mice (Figure4), and because PPARγ agonism by thiazolidinediones (TZDs) enhances peripheral insulin sensitivity (36, 37), we evaluated whether *Aloxe3* enhances peripheral insulin and glucose homeostasis dependent on PPARγ and yet independent of the leptin receptor. Fasting circulating insulin and HOMA-IR were significantly lower in *Aloxe3^db/db^* mice without changes in circulating glucose when compared with *db/db* mice expressing GFP. The reduction in circulating insulin and HOMA-IR were reversed in mice concomitantly treated with the PPARγ inhibitor GW9662, suggesting that *Aloxe3* improves glucose and insulin homeostasis via a PPARγ-dependent mechanism. In contrast with our diet-induced model, however, targeted hepatic *Aloxe3* expression did not reduce circulating lipids in a leptin receptor–deficient mice. Together, the data in our leptin-deficient model elucidate leptin- and PPARγ-dependent functions by *Aloxe3*.

**Figure 9:**
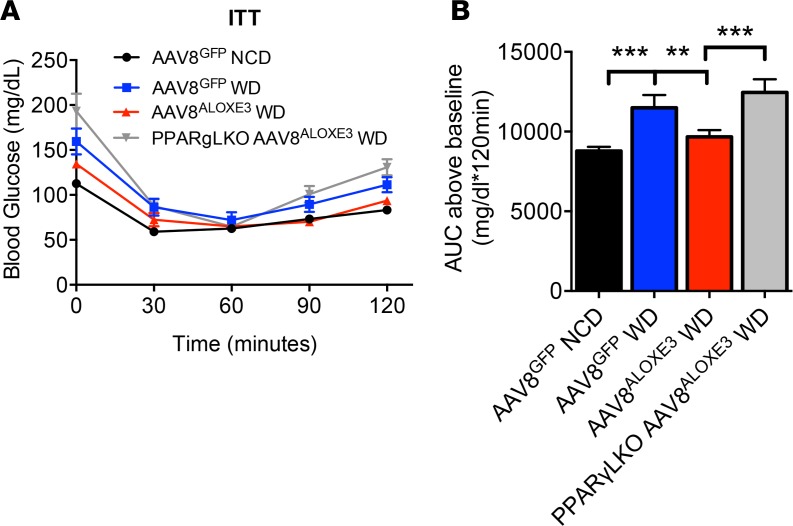
Insulin sensitization by *Aloxe3* requires hepatic PPARγ. (**A**) Insulin tolerance testing in WT or PPARγ-LKO mice expressing hepatic GFP or *Aloxe3* after chow or Western diet. (**B**) Quantification of total area under the glucose time curve. **P < 0.01 and ***P < 0.001 by 2-tailed *t* test with Bonferroni-Dunn post hoc correction between bracketed comparison groups as indicated. Number of mice in each group is: 4, AAV8^GFP^ Chow; 5, AAV8^GFP^ WD; 5, AAV8^ALOXE3^ WD; and 4, PPARγ-LKO AAV8^ALOXE3^ WD.

To gain further specificity regarding the role of hepatocyte PPARγ in *Aloxe3*-enhanced insulin sensitivity, we generated mice harboring a hepatocyte-specific *PPARG* deletion (hereafter referred to as PPARγ-LKO mice) by crossing mice with homozygous floxed *Pparg* alleles with mice expressing Cre recombinase driven by the albumin promoter. Mice were placed on a chow or Western diet (WD) to induce insulin resistance over 12 weeks; they were then subjected to insulin tolerance testing (ITT, Figure 9, A and B). Area under the ITT curve analysis revealed that WD feeding increased AUC in GFP-overexpressing animals, whereas AUC was reduced in *Aloxe3*-overexpressing animals. In striking contrast, WD-fed *Aloxe3*-overexpressing PPARγ-LKO mice had a significantly elevated AUC when compared with WD-fed *Aloxe3*-overexpressing mice harboring WT hepatic PPARγ.

## Discussion

The concept of the fasting and caloric restriction response as a therapeutic to be leveraged in longevity, healthspan, and metabolism from rodents to early primates to humans (1, 38–43) is gaining traction. Already in the clinical realm, caloric restriction improved multiple indices related to aging, cardiovascular disease, and diabetes mellitus (2, 44–46). However, strict and prolonged adherence to caloric restriction, intermittent fasting, or any intensive dietary restriction directed toward weight loss carries with it several practical barriers. Therefore, identifying the therapeutic determinants of the hepatic glucose fasting response opens the promise of novel, targeted, and sustainable treatments against metabolic and other diseases. Here, we show that *Aloxe3* is activated by the adaptive hepatic fasting response and is itself sufficient to enhance insulin sensitivity, increase basal caloric expenditure, and reduce diet-induced weight gain and fat accumulation, hepatic steatosis, and dyslipidemia.

Fully defining the metabolic functions of *Aloxe3* has been previously limited by its basal tissue expression and distribution and by the dramatic phenotype observed in animals and humans deficient for this enzyme. *Aloxe3* was first identified as an epidermal-type lipoxygenase that mediates skin differentiation via hydroperoxide isomerase activity (27, 47). Humans born with a defect in this gene develop ichthyosiform disease, characterized by profound skin barrier dysfunction and transepidermal water loss (28, 30). This was confirmed in murine models of *Aloxe3* deficiency, which succumb to massive dehydration without intervention shortly after birth (32). Subsequent work demonstrated a role for *Aloxe3* in adipocyte differentiation via PPARγ agonism in cultured adipocytes, as well (29). This introduced the possibility that *Aloxe3* has important functions that extend beyond epidermal tissues. However, a potential function in liver was largely ignored, perhaps in part because nonfasting mRNA is relatively low when compared with epidermis and tongue by Northern blot detection (32) and by reverse transcription PCR analysis of murine liver (48). However, we show here that serum and glucose withdrawal, trehalose and lactotrehalose (trehalose analog) treatment in vitro, trehalose feeding, and acute fasting in vivo each robustly induce *Aloxe3* mRNA expression. This raised the prospect that *Aloxe3* mediates part of the physiological hepatic fasting response. We postulate that the observed increased insulin sensitivity in hepatic *Aloxe3*–overexpressing mice reflects an adaptive response that enhances peripheral insulin sensitivity in preparation for a subsequent meal after prolonged fasting. This function would be particularly useful to maximize macronutrient absorption for animals in which meals are few and far between (e.g., intermittently fasting or hibernating animals). Indeed, this teleological rationale has been proposed for other physiological fasting-induced pathways, such as FGF21 signaling (49, 50).

The data herein demonstrate that *Aloxe3* ameliorated the diet-induced obesity and *db/db* diabetic phenotypes, at least in part, via a PPARγ-dependent mechanism — most prominently with regard to the insulin-sensitizing effects of *Aloxe3*. *Aloxe3* overexpression and trehalose treatment in hepatocytes induced the PPARγ ligand 12-KETE at cellular concentrations that appear to be physiologically relevant (19). Indeed, 12-KETE induced sebocyte PPARγ signaling at ~1,500 ng/ml (19), whereas our calculated 12-KETE concentrations in *Aloxe3*-overexpressing hepatocytes (accounting for a hepatocyte volume of roughly 3 fL, femtoliters; ref. 51) indicate concentration of 12-KETE is in the range of 1,000 ng/ml (Figure 2C). In addition, PPARγ blockade by GW9662 administration in isolated hepatocytes and in vivo reversed *Aloxe3*-mediated changes in hepatocyte oxygen consumption rate, insulin resistance, and PGC1α upregulation. Finally, liver-specific PPARγ deletion abrogated *Aloxe3*-enhanced insulin sensitivity. Together, these data introduce a potentially novel hepatic *Aloxe3*–PPARγ axis that improves whole-body insulin sensitivity. These findings are consistent with the previously demonstrated control of peripheral insulin sensitivity via hepatic PPARγ in *ob/ob* mice (34, 35). However, given that GW9662 did not significantly reverse *Aloxe3* effects on hepatic fat accumulation and whole-body thermogenesis, our data indicate that *Aloxe3* exerts both PPARγ-dependent and -independent functions to enhance hepatic and extrahepatic metabolic homeostasis.

It should be noted that genetic disruption of both 12-LOX and 15-LOX in multiple diabetic models largely recapitulates the *Aloxe3*-mediated enhancement of hepatic insulin sensitivity and reduced hepatic steatosis (15–17). Therefore, it is plausible that — in addition to (or perhaps in lieu of) generating the PPARγ ligand 12-KETE — eLOX3 either depletes 12-LOX products 12-HpETE and 12-HETE (Figure 2) to the host’s benefit, or 12-LOX and 15-LOX deletion shunts arachidonic acid down the eLOX3 enzymatic pathway to activate PPARγ. A third distinct possibility is that *Aloxe3* mediates its PPARγ-activating effects independently of upstream lipoxygenases. Three key pieces of data support this possibility. First, hepatocytes are not considered overall to be a significant source of total lipoxygenase activity. This is confirmed by our own data suggesting that basal intrinsic hepatocyte lipoxygenase activity is relatively low — on the order of 1–10 fmol/mg/min (Figure 2B). Second, eLOX3 exhibits latent intrinsic dioxygenase activity, although this catalysis is relatively inefficient when compared with its hydroperoxide isomerase activity (31). Third, in adipose tissue, eLOX3 generated PPARγ agonists in the absence of identifiable upstream lipoxygenases (29). However, regardless of the origin and fate of the lipid intermediaries going through the *Aloxe3* pathway, the net benefit of *Aloxe3* expression involves both PPARγ-dependent and -independent pathways. The species, origin, and precise bioactivity of the lipid intermediaries that underlie these therapeutic effects now arise as a rich topic for further interrogation.

The current study examines therapeutic effects of hepatic *Aloxe3* induction. From a clinical therapeutic perspective, virus-based genetic therapy for many monogenic diseases has already reached clinical care (52). However, this therapeutic approach is not yet fully optimized for polygenic diseases, such as obesity, insulin resistance, and NAFLD (53). Although our physiological, morphometric, and liver histological data did not reveal problematic effects of *Aloxe3* gene overexpression after 12 weeks (Figure 4, Figure 5, and Supplemental Figure 4), a genetic overexpression approach at present is likely to be a more distant clinical therapy when compared with precision small-molecule therapeutics. In addition, although the full extent of *Aloxe3* regulation has yet to be elucidated as a means to leverage *Aloxe3*-enhancing pathways, we demonstrated here that *Aloxe3* is potently upregulated by generalized stimuli such as fasting, trehalose feeding, serum withdrawal, and flavone-class GLUT inhibition. In contrast, canonical fasting intermediates AMPK, PGC1α, FGF21, and PPARα were dispensable for trehalose-induced *Aloxe3* expression. Accordingly, SIRT1 overexpression was insufficient to upregulate *Aloxe3* in vivo. It is therefore intriguing that we were able to achieve enhanced in vitro *Aloxe3* upregulation comparable with viral *Aloxe3* overexpression by treatment with the trehalase-resistant trehalose analogue lactotrehalose (21–23, 54, 55). Our characterization of this glucose-galactose trehalose analogue, and the finding that lactotrehalose exhibits enhanced fasting-mimetic potency, is a critical advance for at least 2 reasons. First, abundant trehalase expression in human gut, liver, brain, kidney, and reproductive tissues raises the possibility that trehalose-based therapies may be limited by trehalase-mediated trehalose degradation. By corollary, trehalase-resistant compounds such as lactotrehalose are postulated to confer enhanced efficacy. Secondly, it is well understood that GLUT8 and GLUT2 (the 2 most highly expressed hepatic glucose transporters; ref. 7) are competitively inhibited by galactose (56–58). In light of the fact that galactose is one of the saccharide moieties that comprises lactotrehalose, the data suggest that altering the saccharide moieties in trehalose modulates potency (and perhaps also selectivity) based on each GLUT’s substrate predilection.

Taken together, although greater detail regarding intermediary regulation of *Aloxe3* is warranted, augmenting *Aloxe3* expression and other components of the hepatic fasting response is now feasible through fasting itself, glucose withdrawal, or trehalose/analogue-class GLUT inhibition. The palatability, and heavy first-pass enterohepatic kinetics (which minimizes peripheral tissue GLUT side effects) indeed make trehalose and its analogues especially attractive candidate nutraceuticals to elicit this response. Future directions should interrogate the fasting response–inducing potential, kinetics, and efficacy of trehalose and its analogs in mitigating metabolic disease (21, 25, 59). In addition, it will be critical to understand the extent to which *Aloxe3* regulation in other tissues is necessary or sufficient to augment tissue-specific adaptive fasting responses. Finally, understanding the full cadre of epoxyalcohols and other lipid products of eLOX3 enzymatic activity, and their specific roles in the mitigating metabolic disease, are of special pharmaceutical and clinical interest (15, 60–63).

In summary, we identified the lipoxygenase Aloxe3 as a potentially novel effector of the hepatic fasting response that is sufficient to augment basal caloric expenditure, and ameliorate insulin resistance, weight gain, and hepatosteatosis. (the meaning I hope to convey is that Aloxe3 does all of the above). The rapidly rising prevalence of each of these major public health problems throughout the Western and developing worlds mandates novel therapeutic pathway generation and leveraging thereof. We assert that further interrogation into how hepatic glucose transport mediates the networked adaptive hepatic fasting response will advance the field toward new and effective human therapy against metabolic disease.

## Methods

### Mouse models and treatment.

All mice were caged in specific pathogen-free barrier housing with 12-hour light-dark cycles and free access to water and rodent chow. For transgenic studies, WT C57B/6J mice and *Lepr^db/db^* mice were obtained directly from the Jackson Laboratory. Upon arrival, mice were randomized to experimental assignments and equilibrated for a minimum of 7 days in the specific pathogen-free vivarium with automated 12-hour light-dark cycles and free access to water and rodent chow. Prior to initiating metabolic measurements. ATG16L1^HM^ mice were a provided by Herbert “Skip” W. Virgin’s laboratory (Washington University School of Medicine) (64). PPARγ-LKO mice were obtained directly from the Jackson Laboratory to the laboratory of David Rudnick (Washington University School of Medicine) and, after propagation, were provided for experimentation.

Adeno-associated viruses overexpressing either GFP or *Aloxe3* under control of the thyroid binding globulin (TBG) promoter were obtained as ready-to-use viral stocks from Vector Biolabs. Viral particles (1 × 10^11^ per animal) were injected via tail vein in 6-week-old mice 10 days prior to dietary initiation or 38 days prior to sacrifice in the genetic obesity model (e.g., 10 days rest period + 28 day dietary stimulus) *Aloxe3* expression was quantified in pilot experiments by qPCR analysis of hepatic tissue, skeletal muscle tissue, and brown and epididymal WAT 10 days after injection and confirmed selective hepatic *Aloxe3* overexpression (not shown).

Antisense oligonucleotides (ASOs) were obtained from IONIS Pharmaceuticals as ready-to-inject oligomers, and they were used precisely as described previously (65). GW9662 was obtained from Cayman Chemicals (catalog 70785). WD is from Harlan Teklad (catalog TD88137); HTF-C diet is from Research Diets (catalog 09100301).

### Lactotrehalose synthesis and purification.

Synthesis and purification of lactotrehalose was carried out precisely as described (66). We confirmed 98%–99% purity by ^1^H-NMR (not shown).

### Lipoxygenase activity assay.

LOX activity was quantified using a lipoxygenase activity assay kit (Biovision, catalog 978-100) per manufacturer instructions.

### Serum analyses.

Fasting blood glucose was measured via glucometer using tail vein blood. For all other serum analyses, submandibular blood collection was performed immediately prior to sacrifice, and serum was separated. Insulin ELISA (MilliporeSigma, catalog EZRMI-13K), TG (Thermo Fisher Scientific, catalog TR22421), cholesterol (Thermo Fisher Scientific, catalog TR13421), and free FA (FFA) (Wako Diagnostics, catalog 999-34691, 995-34791, 991-34891, and 993-35191) quantification was performed using commercially available reagents according to manufacturer’s directions. Albumin levels were quantified using an AMS LIASYS Chemistry Analyzer.

### Hepatic lipids.

Lipids were extracted from ~100 mg hepatic tissue homogenized in 2:1 chloroform/methanol. Extract (0.25%–0.5% of each) was evaporated overnight prior to biochemical quantification of TGs, LDL-C, cholesterol, and FFA using reagents described above, according to manufacturer’s directions.

### Oil Red O staining.

Methanol-fixed frozen sections from WT and transgenic mice were stained according to described protocols (7, 9, 12).

### Body composition analysis and indirect calorimetry.

Body composition analysis was carried out in unanesthetized mice as described (7, 8, 67) using an EchoMRI 3-1 device (Echo Medical Systems) via the Washington University Diabetic Mouse Models Phenotyping Core Facility.

The first 4 hours in the cage were unmeasured in order to allow for acclimation time. Thereafter, the oxygen and CO_2_ consumption and production were quantified for a minimum of 1 light cycle (0601–1800 hours) and for 1 dark cycle (1801–0600 hours). Volume O_2_ (VO_2_), VCO_2_, heat, movement, and respiratory exchange ratio (RER) were automatically calculated using TSE Phenomaster software.

### In vitro metabolism (Seahorse XF96) assays.

Seahorse assays were performed using a Seahorse XF96 analyzer Mito Stress Test Kit (Agilent Technologies) according to manufacturer specifications. Hepatocyte cultures were seeded at 20,000 cells per well prior to assay.

### Immunoblotting.

Immunoblotting was performed as described (67). Anti-GAPDH (catalog 5174) was obtained from Cell Signaling Technologies. LC3B antiserum was obtained from Novus Biologicals (catalog NB-100-2200).

### Indirect calorimetry.

Oxygen consumption, CO_2_ production, RER, and heat production were measured using the Phenomaster system (TSE) via the Washington University Diabetic Mouse Models Phenotyping Core Facility as described (8, 67). Metabolic parameters were documented every 13 minutes.

### Cell cultures and treatment.

Primary murine hepatocytes obtained from WT mice were isolated as described (7, 9–11) and cultured and maintained in regular DMEM growth media (MilliporeSigma, catalog D5796) containing 10% FBS. For in vitro starvation experiments, starved media containing 1 g/l glucose and 0.5% FBS was used. Cultures were lysed in Trizol and subjected to downstream analysis. In vitro genetic knockdown was achieved via siRNA transfection using Lipofectamine 3000 from Invitrogen (catalog L3000015). Trehalose was obtained from MilliporeSigma and was >97% purity by HPLC. Trehalose water (3%, w/v) fed ad libitum was used in all in vivo experiments. Huh7 and HepG2 cell lines were obtained directly from the American Type Culture Collection (ATCC). Human primary hepatocyte cDNAs were obtained directly from Sciencell (catalog 5204).

### qPCR.

qPCR was performed as previously reported (7, 12) with some modifications. Snap-frozen livers or cultured hepatocytes were homogenized in Trizol reagent (Invitrogen, catalog 15596026). RNA isolated according to the manufacturer’s protocol was reverse-transcribed using the Qiagen Quantitect reverse transcriptase kit (Qiagen, catalog 205310). cDNA was subjected to qPCR using the SYBR Green master mix reagent (Applied Biosystems, catalog 4309155). Expression data are depicted as a mean ratio of target gene expression relative to 36B4 (housekeeping gene) expression in each sample, ± SEM. Primer sequences for qPCR are shown in Supplemental Table 1.

### RNA-seq.

RNA-seq was performed by the Washington University Genome Technology Access Center (GTAC). Primary data, processed data, and metadata for these experiments were deposited in the Gene Expression Omnibus (www.ncbi.nlm.nih.gov/geo), with accession number GSE116516.

Library preparation was performed with 10 μg of total RNA with a Bioanalyzer RIN score greater than 8.0. Ribosomal RNA was removed by poly-A selection using Oligo-dT beads (mRNA Direct kit, Invitrogen). mRNA was then fragmented in buffer containing 40 mM tris acetate pH 8.2, 100 mM potassium acetate, and 30 mM magnesium acetate and heated to 94°C for 150 seconds. mRNA was reverse transcribed to yield cDNA using SuperScript III RT enzyme (Invitrogen, per manufacturer’s instructions) and random hexamers. A second strand reaction was performed to yield double-stranded cDNA (ds-cDNA). cDNA was blunt ended, had an A base added to the 3′ ends, and then had Illumina sequencing adapters ligated to the ends. Ligated fragments were then amplified for 12 cycles using primers incorporating unique index tags. Fragments were sequenced on an Illumina HiSeq-3000 using single reads extending 50 bases.

RNA-seq reads were aligned to the Ensembl release 76 top-level assembly with STAR version 2.0.4b. Gene counts were derived from the number of uniquely aligned unambiguous reads by Subread:featureCount version 1.4.5. Transcript counts were produced by Sailfish version 0.6.3. Sequencing performance was assessed for total number of aligned reads, total number of uniquely aligned reads, genes and transcripts detected, ribosomal fraction known junction saturation, and read distribution over known gene models with RSeQC version 2.3.

To enhance the biological interpretation of the large set of transcripts, grouping of genes/transcripts based on functional similarity was achieved using the R/Bioconductor packages GAGE and Pathview. GAGE and Pathview were also used to generate pathway maps on known signaling and metabolism pathways curated by Kyoto Encyclopedia of Genes and Genomes (KEGG).

### Liquid chromatography-tandem mass spectral analysis of eicosanoids.

Eicosanoids (5-HETE, 12-HETE, and 12-KETE) were extracted with 300 μl of 1:1 methanol/water, containing 2 ng deuterated 12-HETE-d_8_ (Cayman Chemical) as the internal standard. This extract (33% of total) was injected; therefore, 0.67 ng represents the total internal standard analyzed. The quantity of eicosanoids calculated based on the peak are the ratio between the analyte and internal standard. Eicosanoid and internal standard analyses were performed using a Shimadzu 20AD HPLC system, and a LeapPAL autosampler coupled to a tandem mass spectrometer (API 4000, API-Sciex). These were operated in negative ion MRM mode. The sample was injected on to a Thermo-Keystone betasil C-18 HPLC column (2 × 100 mm, 3 μm) with mobile phases (A, 5 mM ammonium fluoride in water; B, 100 % acetonitrile). The data processing was conducted with Analyst 1.6.3 (API-Sciex).

### ITT.

ITT was performed precisely as reported previously (8). Briefly, mice were fasted 4 hours prior to i.p. administration of 0.75 U/kg insulin. Glucose was monitored by glucometer (One Touch Ultra, Lifescan) over the course of 2 hours following injection.

### Statistics.

Data were analyzed using GraphPad Prism version 6.0 (RRID:SCR_015807). A *P* value less than 0.05 was defined as statistically significant. Data shown are as mean ± SEM. Unpaired 2-tailed homoscedastic *t* tests with Bonferroni post hoc correction for multiple comparisons were used for all analyses unless otherwise noted in the figure legends. Two-way ANOVA was also used for analyses with 2 independent variables.

### Study approval.

All animal procedures were reviewed and approved by the Washington University School of Medicine Animal Studies Committee.

## Author contributions

BJD conceived and coordinated the study and wrote the paper. CBH, YZ, ALM, HF, MJG, BMS, AIS, and BJD designed, performed, and analyzed the experiments. All authors reviewed the results and approved the final version of the manuscript.

## Supplementary Material

Supplemental data

Supplemental Table 1

## Version 1 08/23/2018

Electronic publication
