# Supplementary material for: Hepatocyte ALOXE3 is induced during adaptive fasting and enhances insulin sensitivity by activating hepatic PPARγ
Source: JCI Insight. 2018 Aug 23;3(16):e120794. doi: 10.1172/jci.insight.120794 (PMC6141168; doi:10.1172/jci.insight.120794)
Supplement: Supplemental data [file jciinsight-3-120794-s010.pdf]

Supplemental Figure 1

A.

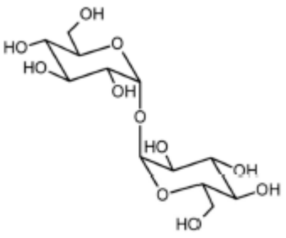

B.

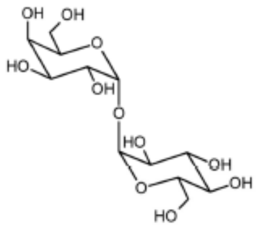

C.

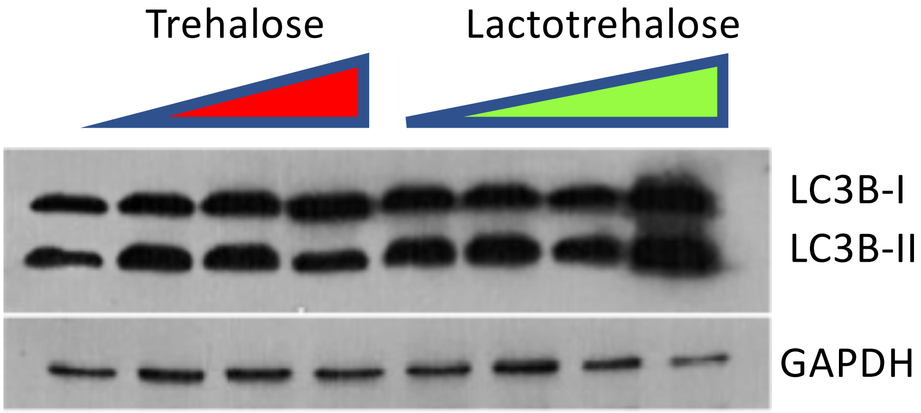

D.

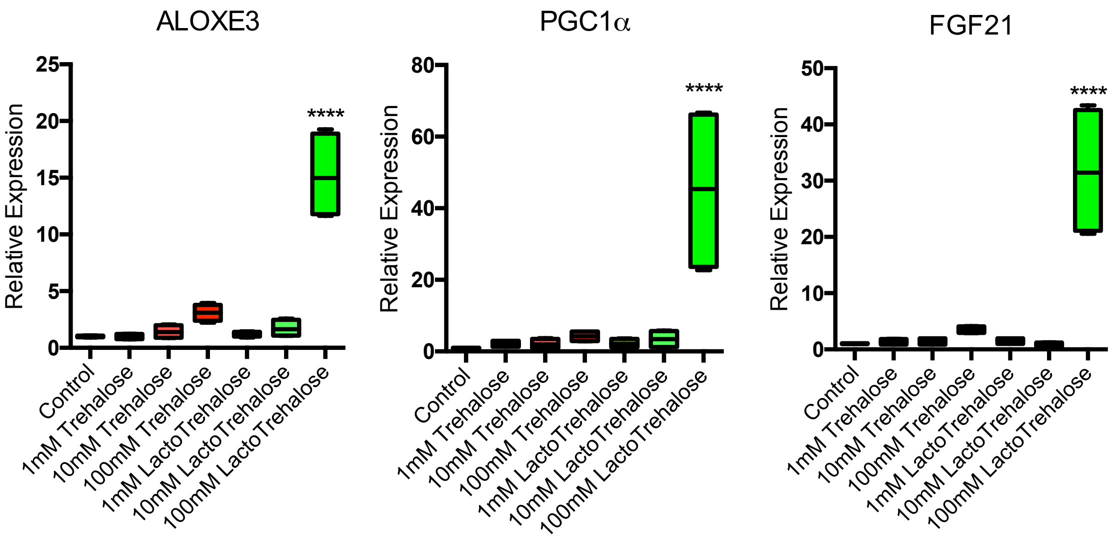

Supplemental Figure 1. Enhanced starvation-induced autophagy and canonical fasting responses by the trehalase-resistant analogue, lactotrehalose. A. Chemical structure of native trehalose. B. Chemical structure of lactotrehalose. C. LC3B immunoblot in lysates derived from isolated primary murine hepatocytes demonstrating LC3B-II accumulation in response to 0, 1mM, 10mM or 100mM trehalose or 0.1mM, 1mM, 10mM or 100mM lactotrehalose for 24h. D. Relative expression of *Aloxe3* and canonical fasting-induced transcripts PGC1α, FGF21 in isolated primary murine hepatocytes treated with increasing dosages of trehalose or lactotrehalose for 24h. n = 4 hepatocyte cultures per group run in 2 independent experiments. ****, P < 0.0001 vs. 100mM Trehalose control by 2-tailed T-Test.

Supplemental Figure 2

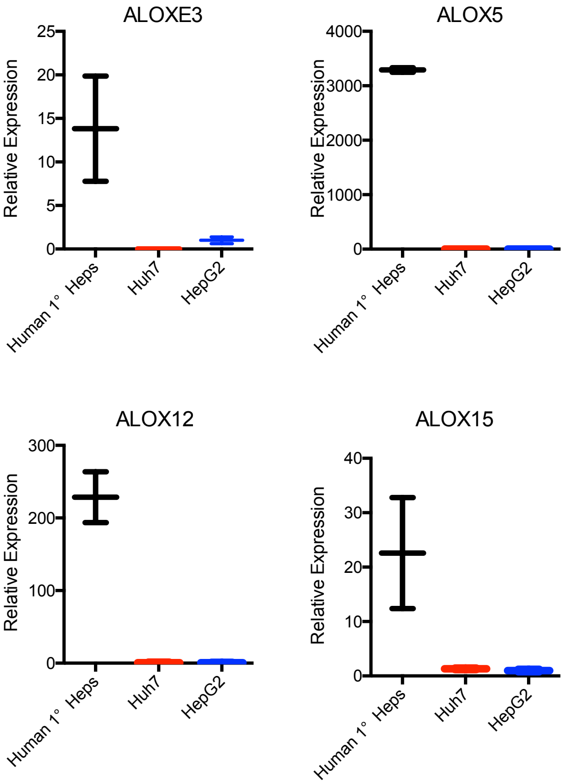

Supplemental Figure 2. Lipoxygenase expression in isolated human hepatocytes. Shown are qPCR data normalized internally to 36B4 expression, plotted as relative to HepG2 lipoxygenase expression, defined as the reference value. For primary human hepatocytes, n = 2. Huh7 and HepG2 n = 4.

Supplemental Figure 3

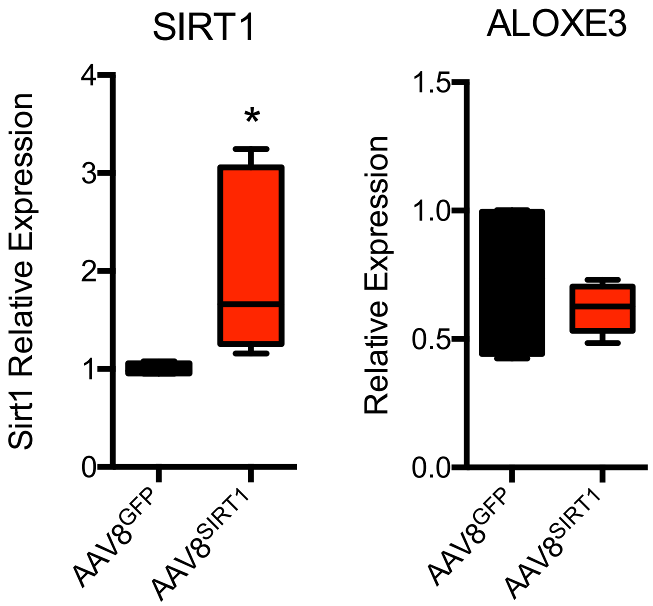

Supplemental Figure 3. Mild SIRT1 overexpression is insufficient to induce hepatic *Aloxe3* in vivo. Mice were injected via tail-vein with AAV8-TBG-SIRT1, sacrificed after 10 days, and hepatic mRNA expression of SIRT1 and ALOXE3 were quantified by qRT-PCR. n = 4 $AAV8^{GFP}$, and 6 $AAV8^{SIRT1}$. *, $P < 0.05$, by 2-tailed T-test versus GFP-expressing controls

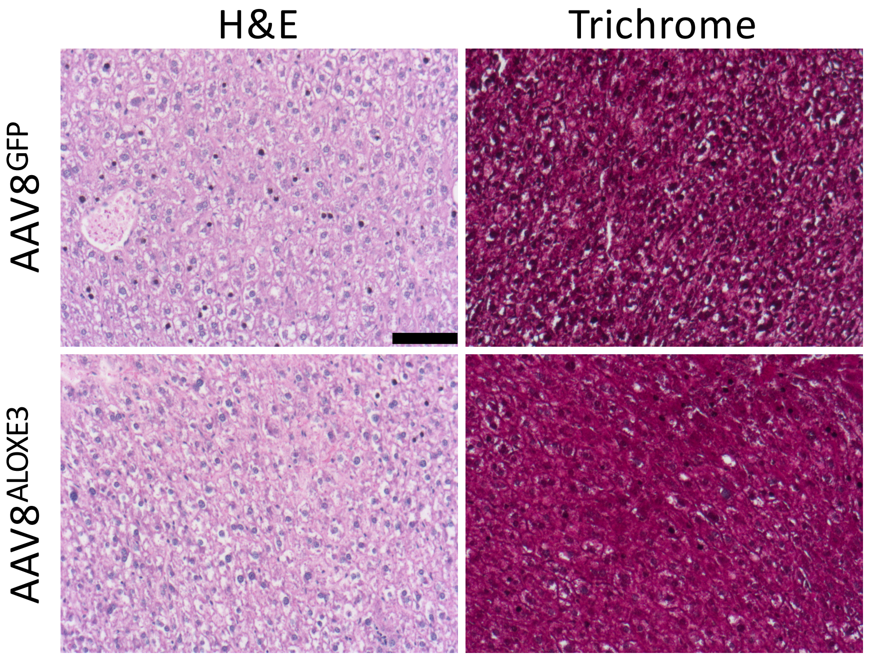

Supplemental Figure 4. No hepatic inflammation or fibrosis in mice overexpressing *Aloxe3* for 12 weeks. Shown are H&E- or Masson's Trichrome-stained liver sections from mice treated with AAV8 encoding empty vector or thyroid binding globulin promoter-driven *Aloxe3*. Scale Bar: 100µm. Data are representative sections analyzed from 5 mice in each treatment group.
