# Supplementary material for: Hepatocyte ALOXE3 is induced during adaptive fasting and enhances insulin sensitivity by activating hepatic PPARγ
Source: JCI Insight. 2018 Aug 23;3(16):e120794. doi: 10.1172/jci.insight.120794 (PMC6141168; doi:10.1172/jci.insight.120794)
Supplement: Supplemental Table 1 [file jciinsight-3-120794-s011.pdf]

Target	Forward Primer Sequence (5’ – 3’)	Reverse Primer Sequence (5’ – 3’)
Aloxe3	ATGGCAGTATATCGGCTGTGT	GCTTCTGCTTAGGGCTTTCAC
Alox5	AGCATGAAAGCAAGGCGCATA	GTACGCATCTACGCAGTTCTG
Alox12	TCCCTCAACCTAGTGCGTTTG	GTTGCAGCTCCAGTTTCGC
Alox15	GGCTCCAACAACGAGGTCTAC	AGGTATTCTGACACATCCACCTT
Fasn	GGAGGTGGTGATAGCCGGTAT	TGGGTAATCCATAGAGCCCAG
Fsp27	GCCCCCATCAGAACAGCGCA	GCCTTCACGTTCAGGCAGCCA
Hnf4a	ATGCGACTCTCTAAAACCCTTG	ACCTTCAGATGGGGACGTGT
Il1b	GCAACTGTTCCTGAACTCAACT	ATCTTTTGGGGTCCGTCAACT
Pck1	GATGGGCATATCTGTGCTGG	CAGCCACCCTTCCTCCTTAG
Ppargc1a	ACACCGCAATTCTCCCTTGT	CGGCGCTCTTCAATTGCTTT
Ppara	TGGTTCCTGGTGCCGATTTA	ACTAGCATCCCACTTAATTATGTATCT
Scd1	ACGCCGACCCTCACAATTC	CAGTTTTCCGCCCTTCTCTTT
Sirt1	TGCTGGCCTAATAGACTTGCA	GCACCGTGGAATATGTAACGA
Tnfa	CAGGCGGTGCCTATGTCTC	CGATCACCCCGAAGTTCAGTAG

Supplemental Table 1. Primer sequences used for qRT-PCR
